# Long-Term Impact of Zinc Oxide Nanoparticles on Differentiation and Cytokine Secretion of Human Adipose-Derived Stromal Cells

**DOI:** 10.3390/ma12111823

**Published:** 2019-06-05

**Authors:** Katrin Radeloff, Andreas Radeloff, Mario Ramos Tirado, Agmal Scherzad, Rudolf Hagen, Norbert H. Kleinsasser, Stephan Hackenberg

**Affiliations:** 1 Department of Otorhinolaryngology, Head and Neck Surgery, Evangelisches Krankenhaus, Carl von Ossietzky-University, 26122 Oldenburg, Germany; andreas.radeloff@uni-oldenburg.de; 2 Department of Otorhinolaryngology, Plastic, Aesthetic and Reconstructive Head and Neck Surgery, Julius-Maximilian-University, 97080 Wuerzburg, Germany; mramos76@yahoo.com (M.R.T.); scherzad_a@ukw.de (A.S.); hagen_r@ukw.de (R.H.); hackenberg_s@ukw.de (S.H.); 3 Department of Otorhinolaryngology, Head and Neck Surgery, Kepler-University, 4021 Linz, Austria; Norbert.Kleinsasser@kepleruniklinikum.at

**Keywords:** zinc oxide, nanoparticles, toxicity, differentiation potential, human adipose-derived stromal cells, stem cells

## Abstract

Zinc oxide nanoparticles (ZnO-NPs) are widely utilized, for example in manufacturing paints and in the cosmetic industry. In addition, there is raising interest in the application of NPs in stem cell research. However, cytotoxic, genotoxic and pro-inflammatory effects were shown for NPs. The aim of this study was to evaluate the impact of ZnO-NPs on cytokine secretion and differentiation properties of human adipose tissue-derived stromal cells (ASCs). Human ASCs were exposed to the subtoxic concentration of 0.2 µg/mL ZnO-NPs for 24 h. After four weeks of cultivation, adipogenic and osteogenic differentiation procedures were performed. The multi-differentiation potential was confirmed histologically and using polymerase chain reaction (PCR). In addition, the gene expression of IL-6, IL-8, vascular endothelial growth factor (VEGF) and caspase 3 was analyzed. Over the course of four weeks after ZnO-NPs exposure, no significant differences were detected in the gene expression of IL-6, IL-8, VEGF and caspase 3 compared to non-exposed cells. The differentiation was also not affected by the ZnO-NPs. These findings underline the fact, that functionality of ASCs is likely to be unaffected by ZnO-NPs, despite a long-term disposition of NPs in the cells, supposing that the starting concentration was safely in the non-toxic range. This might provide important information for single-use nanomedical applications of ZnO-NPs.

## 1. Introduction

Zinc oxide nanoparticles (ZnO-NPs) are one of the most commonly used metal oxide nanoparticles, especially as an ingredient in paints or cosmetic products [1]. Research increasingly focuses on biological and biomedical applications of ZnO-NPs due to their physical and chemical characteristics [2,3]. ZnO-NPs may serve as vehicles for drug delivery [2]. In addition, due to their photocatalytic properties, ZnO-NPs are discussed as new anti-cancer agents in modified photodynamic therapy since they induce increased cytotoxicity in head and neck squamous carcinoma cells (HNSCC) compared to non-malignant cells, even in low-dose application [4]. 

Human adipose tissue-derived stromal cells (hASCs) are a subset of human mesenchymal stem cells (hMSCs) and can easily be harvested in a large amount with low donor-site morbidity [5]. They are characterized by their capacity of differentiation into various mesenchymal lineages and the secretion of various growth factors and cytokines [6,7,8]. Thus, they are applied in wound healing management to improve tissue regeneration and to avoid scar formation [9], as well as in plastic surgery to enhance fat graft survival [9,10,11]. In addition, they migrate into the direction of inflammation or tumor sites. Thus, hMSCs may be suitable as vehicles for antitumor therapeutic agents, such as NPs [12,13]. Despite the wide range of possible applications of hMSCs in regenerative medicine targeting stem cell therapy, guiding, homing and fixing the cells at the desired site are still limiting factors. These challenges can be overcome using NPs [14,15]. In addition, hMSCs represent suitable non-malignant cells for toxicological investigations of NPs under long-term cultivation conditions, since hMSCs can be expanded over several passages without transformation or immortalization [16,17]. 

Information about the toxicity of the NPs, as well as functional impairment of hMSCs, is still incomplete and partially contradictory [1,14,17]. For instance, it is unclear whether superparamagnetic iron oxide nanoparticles inhibit the chondrogenic differentiation of hMSCs [18] or not [19]. Our group previously demonstrated the intracellular accumulation of ZnO-NPs in the cytoplasm of hASCs after a cultivation period of three weeks. The ASCs showed a significant impairment of cell migration after ZnO-NPs exposure, but no alteration of their multi-differentiation potential in histologic analyses [12]. 

The wide range of possible applications of NPs in the stem cell field requires detailed information about the interactions between NPs and human stem cells. Thus, the purpose of the present study was to evaluate the impact of subtoxic concentrations of ZnO-NPs on cytokine expression and multi-differentiation potential of ASCs, four weeks after exposure.

## 2. Results

### 2.1. Characterization of Nanoparticles

According to transmission electron microscopy (TEM), nanoparticles were spherical in shape with a mean diameter of 45–55 nm. Regarding measurements of the dynamic light scattering, the average diameter of particle aggregates was 120.68 nm with a polydispersity index of 0.136 and a zeta potential of −11.2 mV. 

### 2.2. Detection of ZnO-NPs in ASCs Using TEM

**Figure 1:**
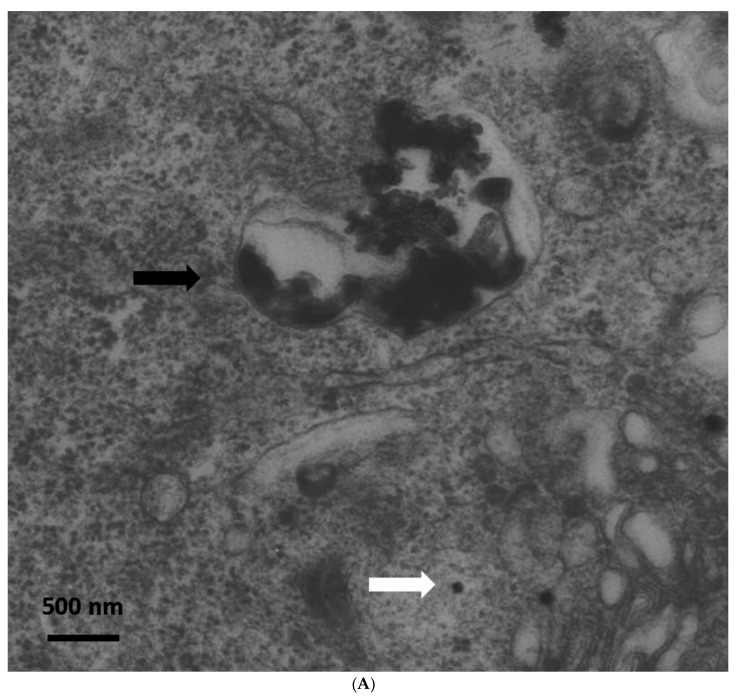

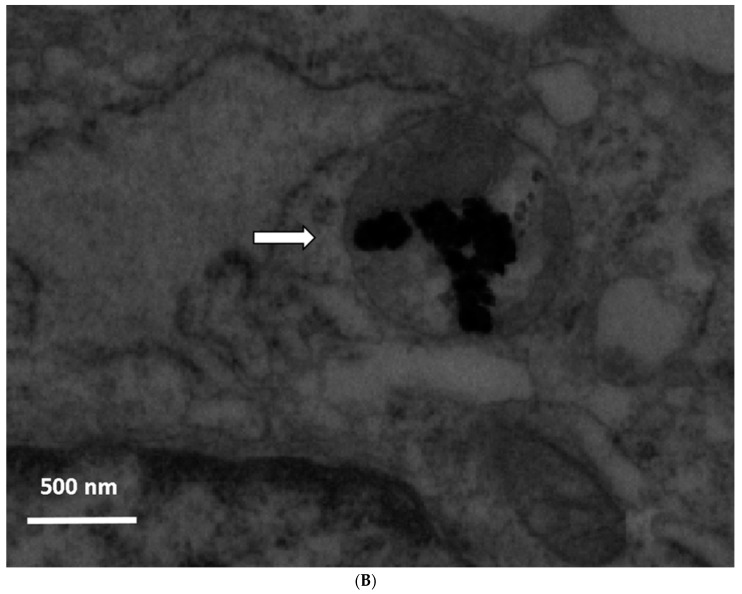
(**A**) Transmission electron microscopy (TEM) photograph of intracellular distribution of zinc oxide nanoparticles (ZnO-NPS) in human adipose tissue-derived stromal cells (ASCs) one week after 24 h of exposure. The black arrow indicates a vesicle containing conglomerates of ZnO-NPs. The white arrow indicates single particles in the cytoplasm (scale bar represents 500 nm). (**B**) TEM photograph of agglomerated ZnO-NPs within a lysosome (indicated by the white arrow) in human ASCs, two weeks after exposure. The scale bar represents 500 nm.

Immediately after exposure, no ZnO-NPs were detectable within the cells. Particle aggregates were found attached to the cell membrane, however, no inclusion of particles into the cells was observed. Clearly, the intracellular presence of ZnO-NPs within the exposed ASCs could be determined at all other time points (one, two, three, and four weeks after exposure). After one week, TEM patterns still showed some intracellular vesicles in the cytoplasm containing small zinc oxide particles in the exposed ASCs (Figure 1). After two weeks and at later time points, no more particle-containing vesicles were found. Instead, free particle aggregates were seen in the cytoplasm or rarely in organelles, but preferably lysosomes (Figure 1B). No inclusion into the nucleus was assessed.

### 2.3. Cytotoxicity and Viability of ASCs

**Figure 2:**
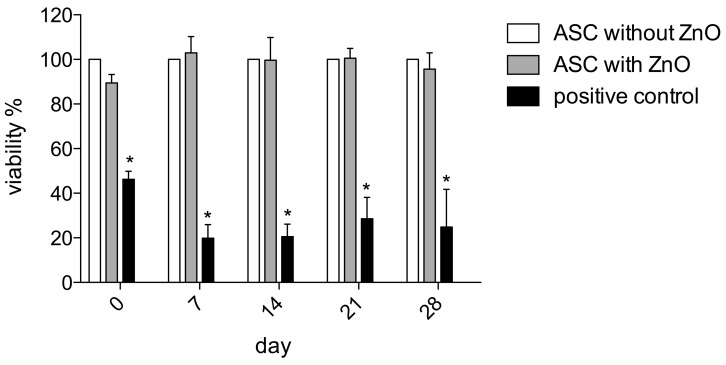
The MTT assay was performed to assess the impact on ASCs’ proliferation and possible cytotoxic effects after ZnO-NPs exposure. The analyses revealed no impairment of viability compared to the unexposed controls immediately after exposure and 7, 14, 21 as well as 28 days after the procedure. The mean extinction values were averaged from 8 wells per patient and normalized to the respective values of the unexposed ASCs of the same patient. The value of unexposed ASCs was equalized to a viability of 100% per patient. Significance is indicated by asterisks (* *p* < 0.001).

The MTT assay was performed to assess the long-term impact on the proliferation of the ASCs and possible cytotoxic effects after ZnO-NPs exposure. The analyses revealed no impairment of viability compared to the unexposed controls immediately after exposure and seven days, 14 days, 21 days, as well as 28 days after the procedure (Figure 2). 

### 2.4. Multilineage Differentiation Potential

#### 2.4.1. Histology

**Figure 3:**
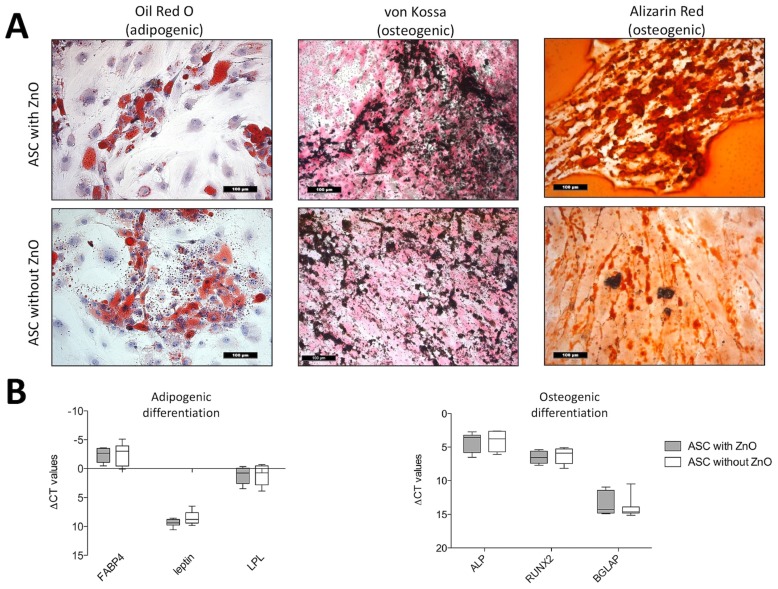
(**A**) Histologic analyses of adipogenic and osteogenic differentiation in ZnO-NPs exposed and unexposed ASCs: Oil Red O-stained intracellular lipid vacuoles could be detected in both exposed and unexposed cells after three weeks of adipogenic differentiation. Deposition of extracellular calcium deposits was detected in exposed and unexposed cells using Alizarin Red (red nodules) and von Kossa stain (black nodules) in both groups after osteogenic differentiation. Magnification ×200 in all figures; scale bar represents 100 µm. (**B**) Quantitative analyses of the multilineage differentiation (real-time PCR): There were no differences in the expression of the adipogenic marker genes FABP4, leptin and LPL and the osteogenic marker genes alkaline phosphatase, RUNX-2 and BGLAP (osteocalcin) between exposed and unexposed ASCs. Box-whisker plots show the median, 1st quartile, and 3rd quartile, as well as the minimal and maximal values of ∆CT (values normalized to GAPDH).

Comparing the ZnO-NPs-exposed and unexposed ASCs, there were no remarkable differences apparent in the histological patterns of the differentiation assays. Typical intracellular lipid vacuoles were detected in both groups after adipogenic differentiation using the Oil Red O stain (Figure 3A). The ZnO-NPs-exposed and unexposed ASCs, which were maintained in expansion medium, showed no intracellular lipid vacuoles (not shown). The deposition of extracellular calcium after the osteogenic differentiation of the ZnO-NPs-exposed and unexposed cells was confirmed using the Alizarin Red and von Kossa stains (Figure 3A), whereas the negative controls revealed no extracellular matrix deposition (not shown). 

#### 2.4.2. Real Time-PCR Analyses

In addition, the differentiation potential was confirmed quantitatively by the measurement of gene expression levels of fatty acid binding protein 4 (FABP4), leptin, and lipoproteinlipase (LPL), which are specific marker genes for adipogenic differentiation and alkaline phosphatase (ALP), Runt-related transcription factor 2 (RUNX-2) and osteocalcin (bone gamma-carboxylglutamate protein, BGLAP), which indicate osteogenic differentiation. Both ZnO-NPs-exposed and unexposed cells, presented no differences in gene expression (Figure 3B). 

### 2.5. Gene Expression of IL- 6, IL-8, Vascular Endothelial Growth Factor A (VEGF A) and Caspase 3

**Figure 4:**
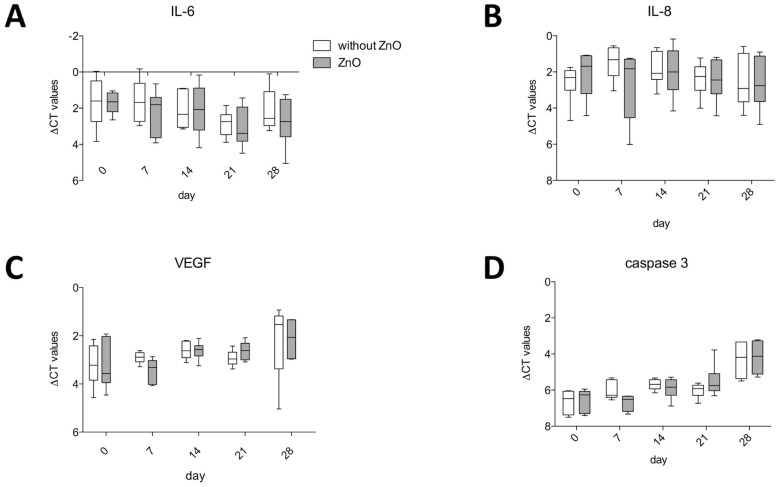
ZnO-NP-exposed and unexposed ASCs presented no differences in the gene expression values of IL-6 (**A**), IL-8 (**B**), VEGF (**C**) and caspase 3 (**D**). Box-whisker plots show the median, 1st quartile, and 3rd quartile, as well as the minimal and maximal values of ∆CT (values normalized to GAPDH).

The ZnO-NPs-exposed and unexposed ASCs presented no differences in the gene expression values of IL-6 (Figure 4A), IL-8 (Figure 4B), VEGF (Figure 4C) as well as caspase 3 (Figure 4D).

## 3. Discussion

ZnO-NPs are widely used as an additive to paints and cements, as well as in the pharmaceutical and cosmetic industries. ZnO-NPs are an important ingredient of sunscreens. Consumers, as well as employees in the chemical industry, come into contact with ZnO via skin and airway exposure, respectively [1]. While the stratum corneum of intact skin may sufficiently protect the organism against NPs, injuries or skin lesions due to chronic skin disease or sunburn may allow the NPs to enter deeper layers of the epidermis with their proliferating cells [1,20,21]. Recently, after topical application of ZnO-NPs in vivo, an accumulation within the hair follicles with consecutive apoptosis of the hair follicle stem cells (HFSCs) was described. Additional in vitro studies revealed DNA damage, impairment of the differentiation potential of the HFSCs and altered expression of marker genes for apoptosis, differentiation and cell communication [22]. 

Besides their common use in consumer products, various applications of NPs in stem cell research and medicine are discussed [14]. Magnetic nanoparticles can be used for tracking and guiding stem cells to their desired site or as a vector for targeted delivery of biotherapeutics [14,23]. In addition, hMSCs may be suitable as vehicles for antitumor therapeutic NPs [12,13]. As presented in a previous study, the exposure of ZnO-NPs to a head and neck cancer cell line induced a G2/M phase arrest under subtoxic conditions. ZnO-NPs should be evaluated regarding their potential to sensitize cells to ionizing radiation. In this context, stem cells could be an interesting carrier for particles in order to stay within the tumor formation due to a high migration and adhesion of stem cells towards tumor tissue [24]. However, the impact of NPs exposure and the intracellular accumulation of NPs long-term after exposure [17,22] on functional capabilities of stem cells remain unclear and partially controversial [12,18,19,22]. Thus, it was the aim of the present study to provide additional information about the interaction between ZnO-NPs and human adipose-derived stromal cells (hASCs) with the focus on functional aspects.

During four weeks after 24 h of exposure to a subtoxic concentration of ZnO-NPs, the viability of the hASCs and the gene expression of IL-6, IL-8, VEGF, and caspase 3 was determined every week. In addition, the present study evaluated the differentiation potential of human ASCs after ZnO-NP exposure. Our evaluation was not only based on qualitative visual observations using the histological patterns, but also on the quantitative analysis of specific marker gene expression for adipogenic and osteogenic differentiation using real-time PCR. 

All experiments were performed using subtoxic concentrations, as shown by preserved viability of the ASCs after 24 h of exposure and after seven, 14, 21 and 28 days, as confirmed by the MTT assay. After differentiation under specific medium conditions for three weeks, no differences between ZnO-NPs-exposed ASCs and non-exposed cells were apparent in the histological patterns. Gene expression of specific adipogenic and osteogenic marker genes did not significantly differ in both groups. Others described alterations of differentiation marker gene expression in HFSCs after exposure to ZnO-NPs, whereas a distinct concentration and application of ZnO-NPs, as well as a different cell type, were used for the experiments [22]. In the current study, ASCs underwent the multi-differentiation procedures four weeks after exposure to ZnO-NPs, with persisting accumulation of ZnO-NPs within the ASCs. Nevertheless, recovery time after the exposure procedure may facilitate certain repair processes within the exposed cells. However, histologic analyses previously performed did not reveal impaired multi-differentiation potential of the hASCs, which underwent the differentiation procedures immediately after 24 h of ZnO-NPs exposure. However, an impairment of migration capability was observed for exposed hASCs in this study [12]. During differentiation of hMSCs, ZnO-NPs show reduced toxicity on hMSCs, which may be due to a slower proliferation of the differentiated cells [17]. Thus, a decreased compromising effect of ZnO-NPs during the differentiation procedure can be assumed. 

Regarding the gene expression of IL-6, IL-8, and VEGF, no significant differences could be determined between the ZnO-NPs-exposed ASCs and the controls. This is contrary to results of our previous study with silver nanoparticles (Ag-NPs) [25] and the observation of others [26], who demonstrated an increased secretion of IL-8 as an indicator for stem cell activation in vascular endothelial cells after short-term ZnO-NPs exposure. Besides the mediation of stem cell activation, IL-6 and IL-8 are also well-known chemotactic cytokines for the attraction of neutrophils, eosinophils, and T lymphocytes [27]. Elevated levels of these cytokines may induce inflammation in several tissue models after NPs exposure. Such inflammatory potential was already demonstrated for ZnO-NPs [28]. In addition, ZnO-NPs are suspected to induce a chronic pro-inflammatory milieu in the human upper aerodigestive tract [29]. Although NPs were intracellularly trapped in the ASCs over the whole period of observation (four weeks), the cytokine gene expression was not affected. The main difference to other studies, is that we applied a safe and surely non-toxic amount of NPs instead of borderline concentrations, as done in the other studies. Thus, this finding clearly underlines the fact, that the functionality of the cells is likely to be unaffected by ZnO-NPs at low concentrations. In addition, a long-term disposition of NPs in the cells does not influence their functionality, supposing that the starting concentration was safely in the non-toxic range. It is well-known, that the shape and size of the nanoparticles critically influence their toxicity [1,30,31,32,33]. In the current study, we chose one representative nanoparticle size, since our experiments focused on the effect of time on toxicity and functionality. However, physical properties like shape and size must be assessed as well, since data on nanotoxicology of different sized ZnO-NPs in ASCs are lacking. Theories of ongoing intracellular zinc ion release from NP-aggregates is not supported by our findings. This might be an important information for single-use applications of ZnO-NPs on nanomedical approaches. Cells with a short life span will probably release particles during apoptosis, so they will be removed by dendritic cells or macrophages. However, long-living cells, like stem cells, are likely to contain nanoparticles over a long time. In the opinion of the authors, the application of ZnO-NPs under subtoxic concentrations will be safe, even if NPs remain in the cells over a long period. However, repetitive exposure to such low concentrations will surely lead to further NPs accumulation in the cells and studies using repeated NPs exposures are especially interesting in long-living cell types.

## 4. Material and Methods

### 4.1. Chemicals and Their Characterization

The ZnO particle powder (10 mg, <100 nm, purity 99.6%, specific surface area 15–25 m^2^/g; Sigma-Aldrich, St. Louis, MO, USA) was suspended in 870 µL of aqua bidest water and sonicated (Bandelin, Sonopuls HD 60, Berlin, Germany) using a continuous mode. The dispersion was stabilized and neutralized according to Bihari et al. [34], as described previously [35] using 30 µL of 1.5 mg/mL bovine serum albumin (BSA) and 100 µL of 10× concentrated phosphate buffered saline (PBS). This 10 mg/mL stock suspension was diluted with Dulbecco’s Modified Eagle medium (DMEM; Gibco Invitrogen, Karlsruhe, Germany) in order to reach a concentration of 0.2 µg/mL. Dynamic light scattering (Malvern Instruments Ltd., Herrenberg, Germany) was used to evaluate the size distribution of particle aggregates and the zeta potential.

### 4.2. Detection of ZnO-NPs in ASCs Using TEM

To study the ultrastructure of intracellular particle distribution, as well as to quantify particle-containing cells during expansion, TEM was performed as described previously [29]. The ZnO-NPs-exposed and unexposed ASCs were immediately analyzed (< 30 min) after the exposure, as well as after seven, 14, 21 and 28 days. Pellets containing ASCs of three patients each were fixed for 30 min in a solution of 0.1 M sodium cacodylate buffer (pH 7.2), 2.5% glutaraldehyde and 2% formaldehyde. The pellets were post-fixed with 2% osmium tetroxide in 50 mM sodium cacodylate buffer (pH 7.2) for 2 h at 4 °C. Afterwards, staining was performed overnight with 0.5% aqueous uranyl acetate. The specimens were dehydrated, embedded in epoxy resin (Epon 812), cut into ultrathin sections of 60 nm thickness and examined on a Zeiss transmission electron microscope EM 900 (Carl Zeiss AG, Oberkochen, Germany) at the Division of Electron Microscopy (group of Prof. Dr. Krohne, Theodor-Boveri-Institute, University of Wuerzburg). The photographic negatives were digitalized by scanning.

### 4.3. Isolation and Cell Culture of Human Adipose Tissue-Derived Stromal Cells (hASCs)

Human ASCs were used for the present study, which was approved by the Ethics Board of the University Clinic of Wuerzburg (grant # 72/06). The ASCs were isolated from the adipose tissue of 6 healthy donors with their informed consent.

The isolation procedure was previously described [16]. Liposuction material was transferred from the operation theatre under sterile conditions and washed with PBS plus 1% penicillin/streptomycin (P/S; Biochrom AG, Berlin, Germany). Subsequently, the adipose tissue was enzymatically digested with Collagenase P (Roche Diagnostics) for 3 h at 37 °C under continuous shaking. After centrifugation and discarding the supernatant, including the adipose tissue, erythrocyte lysis buffer (154 mM ammonium chloride (NH_4_Cl), 10 mM potassium bicarbonate (KHCO3), 0.1 mM ethylenediaminetetraacetic acid (EDTA)) was added for 10 min to eliminate the erythrocytes. After another centrifugation and washing step, the cells were plated in culture flasks and maintained at 37 °C in expansion medium (DMEM-EM) consisting of DMEM containing 1% P/S and 10% fetal calf serum (FCS; Linaris, Wertheim-Bettingen, Germany). The isolated and plated cells were defined as passage 0. The medium was replaced every third day during expansion. The cell morphology and proliferation was evaluated by microscopy (LEICA DMI 4000B Inverted Microscope, Leica Microsystems, Wetzlar, Germany). The cells were detached with 0.25% trypsin containing 1mM EDTA (Gibco Invitrogen), when they reached 80% confluency. After counting (Casy^®^ Technologies, Innovatis AG, Reutlingen, Germany), 1 × 10^6^ hASCs/mL were frozen in cryopreservation medium (80% FCS, 10% DMEM and 10% dimethylsulfoxide [DMSO]). For the following analyses, the ASCs were thawed, resuspended in DMEM-EM and seeded in culture flasks at a density of 2000 cells/cm^2^. They were defined as passage 1. After reaching 80% confluency, the hASCs were detached (passage 2) and used for the following experiments. 

### 4.4. Exposure of hASCs to ZnO-NPs

Human ASCs of passage 2 were exposed to ZnO-NPs with a final concentration of 0.2 µg/mL. After 24 h of exposure, the medium was removed and the hASCs were extensively washed with PBS. Immediately afterwards, the viability of the hASCs was evaluated using the MTT [3-(4,5-dimethylthiazol-2-yl)-2,5-diphenyl tetrazolium bromide] colorimetric staining method [36,37]. The remaining cells were resuspended in expansion medium and cultured for 4 weeks without any additional exposure to ZnO-NPs during this time course. 

### 4.5. Cytotoxicity and Viability of hASCs

The MTT assay [36,37] was performed to assess hASCs’ viability and possible cytotoxic effects immediately after removing the ZnO-NP-containing medium and after seven, 14, 21 and 28 days of culture. Untreated hASCs served as negative controls. Human ASCs treated with 200 µM tert-butylhydroperoxide (t-BHP; Luperox^®^ TBH70X; Sigma-Aldrich, St. Louis, MO, USA) served as positive controls. The cells were seeded in 96-well plates at a density of 1 × 10^4^ hASCs per well. Eight wells were seeded for each of the six patients at each time point. All of the plates were incubated with 100 µl of MTT solution (1 mg/ml) at 37 °C with 5% CO_2_ for 4 h. Subsequently, the MTT solution was replaced by 100 µl isopropanol for 1 h. The color conversion was measured using a multiplate reader (Titertek Multiskan PLUS MK II, Labsystems, Helsinki, Finland) at a wavelength of 570 nm. The mean extinction values were averaged from 8 wells per patient and normalized to the respective values of the unexposed ASCs of the same patient. The value of unexposed ASCs was equalized to a viability of 100% per patient. Viability of exposed cells and positive control was indicated as a percentage of the viability of the unexposed controls.

### 4.6. Multilineage Differentiation Potential

In order to determine a possible impairment of the multilineage differentiation potential of the hASCs due to ZnO-NPs exposure, adipogenic and osteogenic differentiation procedures were performed, as previously described [30]. For adipogenic differentiation, DMEM containing 1% P/S and 10% FCS supplemented with 1 µg/ml insulin, 10 µM dexamethasone, 100 µM indomethacin and 500 µM 1-methyl-3-isobutylxanthine according to Pittenger et al. [38], modified by Nöth et al. [39], was used for adipogenic differentiation. DMEM—containing 1% P/S and 10% FCS supplemented with 100 nM dexamethasone, 10 mM ß-glycerophosphate and 50 µg/ml ascorbic acid—was used for osteogenic differentiation, according to Jaiswal et al. [40]. Human ASCs of the six patients were used for the multilineage differentiation studies four weeks after the exposure procedure (hASCs of passage 6). Human ASCs were maintained in the defined media for three weeks and the medium was replaced every other day. The adipogenic and osteogenic differentiation was confirmed qualitatively using histology and quantitatively using real time-polymerase chain reaction (PCR) analyses. 

#### 4.6.1. Histology

Human ASCs, which were exposed to ZnO-NPs, as well as unexposed controls, were seeded at a density of 2 × 10^4^ cells/cm^2^ in 4-wells (Greiner Bio-One GmbH, Frickenhausen, Germany). 

To show intracellular lipid vacuoles after adipogenic differentiation, the Oil Red O stain was used. Von Kossa staining revealed extracellular mineral deposition after osteogenic differentiation by the presence of black nodules. In addition, extracellular calcium deposits were stained red using the Alizarin Red solution.

#### 4.6.2. PCR Analyses

Adipogenic and osteogenic differentiation was confirmed by the measurement of lineage-specific gene expression. Human ASCs, which were exposed to ZnO-NPs, as well as unexposed controls, were seeded at a density of 1 × 10^5^ cells/cm^2^ in 6-wells (Greiner Bio-One GmbH, Frickenhausen, Germany). Total-RNA was extracted using the RNeasy Mini Kit (Qiagen, Hilden, Germany) and was dissolved in nuclease-free water afterward. After reverse transcription using the High Capacity RNA-to-cDNA Master Mix (Applied Biosystems, Darmstadt, Germany), analyses were performed on a real-time PCR device (Applied Biosystems), with standard Taqman^®^ assays (Applied Biosystems) using a cDNA input equivalent of 50 ng cDNA per replicate. Fatty acid binding protein 4 (aP2; NM_001442.2), lipoproteinlipase (LPL; NM_000237.2) and leptin (NM_002303.5) were used as specific genes for adipogenic differentiation. In addition, alkaline phosphatase (ALP; NM_000478.4), bone gamma-carboxylglutamate protein (BGLAP, osteocalcin; NM_199173.4) and Runt-related transcription factor 2 (Runx-2/cbfa-1; NM_004348.3) expression was quantified for osteogenic differentiation. Relative quantification was performed and presented as values (∆CT values) normalized to the gene expression of the housekeeping gene GAPDH (NM_002046.3). The gene expression values of the ZnO-NPs-exposed and unexposed hASCs were compared. 

### 4.7. Gene Expression of Interleukin (IL-) 6, IL-8, VEGF A and Caspase 3

The gene expression of interleukin (IL-6 (NM_000600.3), IL-8 (NM_00584.3), vascular endothelial growth factor A (VEGF A; NM_001025366.2) and caspase 3 (CASP3; NM_004346.3) was measured by real-time PCR analyses, as described above. The exposed and unexposed hASCs were harvested immediately after 24 h of ZnO-NPs exposure and after 7, 14, 21 and 28 days of culture. The relative quantification values (∆CT values) were normalized to the gene expression of the housekeeping gene GAPDH (NM_002046.3). The gene expression values of the ZnO-NPs-exposed and unexposed hASCs were compared.

### 4.8. Statistical Analyses

GraphPad 5 (Graphpad Software, La Jolla, CA, USA) was used for all graphs and statistical analyses. A two-way ANOVA with the Bonferroni posthoc test was used for the analysis of the MTT assay (Figure 2) and the gene expression analyses of IL-6, IL-8, VEGF, and caspase 3 (Figure 4). For comparison of gene expression of the ZnO-NPs-exposed and unexposed hASCs after multilineage differentiation procedures, the unpaired *t*-test was used when Gaussian distribution could be confirmed (Figure 3). Otherwise, the Mann–Whitney U test was applied (Figure 3). Significance is indicated in the figures by asterisks. The results were mostly charted using Boxplots. The box shows the median, the 1st quartile, and the 3rd quartile, and the whiskers represent the minimal and maximal values. The columns show the mean and standard error of the mean (SEM).

## References

[1] Scherzad A., Meyer T., Kleinsasser N., Hackenberg S. (2017). Molecular Mechanisms of Zinc Oxide Nanoparticle-Induced Genotoxicity Short Running Title: Genotoxicity of ZnO NPs. Materials.

[2] Rasmussen J.W., Martinez E., Louka P., Wingett D.G. (2010). Zinc oxide nanoparticles for selective destruction of tumor cells and potential for drug delivery applications. Expert Opin. Drug Deliv..

[3] Ramos A.P., Cruz M.A.E., Tovani C.B., Ciancaglini P. (2017). Biomedical Applications of Nanotechnology. Biophys. Rev..

[4] Hackenberg S., Scherzed A., Kessler M., Froelich K., Ginzkey C., Koehler C., Burghartz M., Hagen R., Kleinsasser N. (2010). Zinc oxide nanoparticles induce photocatalytic cell death in human head and neck squamous cell carcinoma cell lines in vitro. Int. J. Oncol..

[5] Oedayrajsingh-Varma M.J., van Ham S.M., Knippenberg M., Helder M.N., Klein-Nulend J., Schouten T.E., Ritt M.J., van Milligen F.J. (2006). Adipose tissue-derived mesenchymal stem cell yield and growth characteristics are affected by the tissue-harvesting procedure. Cytotherapy.

[6] Zuk P.A., Zhu M., Ashjian P., De Ugarte D.A., Huang J.I., Mizuno H., Alfonso Z.C., Fraser J.K., Benhaim P., Hedrick M.H. (2002). Human adipose tissue is a source of multipotent stem cells. Mol. Biol. Cell.

[7] Froelich K., Hagen R., Kleinsasser N. (2014). Adipose-derived stromal cells (ASC)—Basics and therapeutic approaches in otorhinolaryngology. Laryngorhinootologie.

[8] Zielins E.R., Luan A., Brett E.A., Longaker M.T., Wan D.C. (2015). Therapeutic applications of human adipose-derived stromal cells for soft tissue reconstruction. Discov. Med..

[9] Si Z., Wang X., Sun C., Kang Y., Xu J., Wang X., Hui Y. (2019). Adipose-derived stem cells: Sources, potency, and implications for regenerative therapies. Biomed. Pharmacother..

[10] Matsumoto D., Sato K., Gonda K., Takaki Y., Shigeura T., Sato T., Aiba-Kojima E., Lizuka F., Inoue K., Suga H. (2006). Cell-assisted lipotransfer: Supportive use of human adipose-derived cells for soft tissue augmentation with lipoinjection. Tissue Eng..

[11] Yoshimura K., Sato K., Aoi N., Kurita M., Inoue K., Suga H., Eto H., Kato H., Hirohi T., Harii K. (2008). Cell-assisted lipotransfer for facial lipoatrophy: Efficacy of clinical use of adipose-derived stem cells. Dermatol. Surg..

[12] Hackenberg S., Scherzed A., Technau A., Froelich K., Hagen R., Kleinsasser N. (2013). Functional Responses of Human Adipose Tissue-Derived Mesenchymal Stem Cells to Metal Oxide Nanoparticles In Vitro. J. Biomed. Nanotechnol..

[13] Roger M., Clavreul A., Venier-Julienne M.C., Passirani C., Sindji L., Schiller P., Montero-Menei C., Menei P. (2010). Mesenchymal stem cells as cellular vehicles for delivery of nanoparticles to brain tumors. Biomaterials.

[14] El-Sadik A.O., El-Ansary A., Sabry S.M. (2010). Nanoparticle-Labeled Stem Cells: A Novel Therapeutic Vehicle. Clin. Pharmacol..

[15] Kang H.J., Kim J.Y., Lee H.J., Kim K.H., Kim T.Y., Lee C.S., Lee H.C., Park T.H., Kim H.S., Park Y.B. (2012). Magnetic bionanoparticle enhances homing of endothelial progenitor cells in mouse hindlimb ischemia. Korean Circ. J..

[16] Froelich K., Mickler J., Steusloff G., Technau A., Ramos Tirado M., Scherzed A., Hackenberg S., Radeloff A., Hagen R., Kleinsasser N. (2013). Chromosomal aberrations and deoxyribonucleic acid single-strand breaks in adipose-derived stem cells during long-term expansion in vitro. Cytotherapy.

[17] Ickrath P., Wagner M., Scherzad A., Gehrke T., Burghartz M., Hagen R., Radeloff K., Kleinsasser N., Hackenberg S. (2017). Time-Dependent Toxic and Genotoxic Effects of Zinc Oxide Nanoparticles after Long-Term and Repetitive Exposure to Human Mesenchymal Stem Cells. Int. J. Environ. Res. Public Health..

[18] Kostura L., Kraitchman D.L., Mackay A.M., Pittenger M.F., Bulte J.W. (2004). Feridex labeling of mesenchymal stem cells inhibits chondrogenesis but not adipogenesis or osteogenesis. NMR Biomed..

[19] Heymer A., Haddad D., Weber M., Gbureck U., Jakob P.M., Eulert J., Nöth U. (2008). Iron oxide labelling of human mesenchymal stem cells in collagen hydrogels for articular cartilage repair. Biomaterials.

[20] Cross S.E., Innes B., Roberts M.S., Tsuzuki T., Robertson T.A., McCormick P. (2007). Human Skin Penetration of Sunscreen Nanoparticles: In-Vitro Assessment of a Novel Micronized Zinc Oxide Formulation. Skin Pharmacol. Physiol..

[21] Monteiro-Riviere N.A., Wiench K., Landsiedel R., Schulte S., Inman A.O., Riviere J.E. (2011). Safety evaluation of sunscreen formulations containing titanium dioxide and zinc oxide nanoparticles in UVB sunburned skin: An in vitro and in vivo study. Toxicol. Sci..

[22] Ge W., Zhao Y., Lai F.N., Liu J.C., Sun Y.C., Wang J.J., Cheng S.F., Zhang X.F., Sun L.L., Li L. (2017). Cutaneous Applied Nano-ZnO Reduce the Ability of Hair Follicle Stem Cells to Differentiate. Nanotoxicology.

[23] Mok H., Zhang M. (2013). Superparamagnetic iron oxide nanoparticle-based delivery systems for biotherapeutics. Expert Opin. Drug Deliv..

[24] Moratin H., Scherzad A., Gehrke T., Ickrath P., Radeloff K., Kleinsasser N., Hackenberg S. (2018). Toxicological Characterization of Zno Nanoparticles in Malignant and Non-Malignant Cells. Environ. Mol Mutagen..

[25] Hackenberg S., Scherzed A., Kessler M., Hummel S., Technau A., Froelich K., Ginzkey C., Koehler C., Hagen R., Kleinsasser N. (2011). Silver nanoparticles: Evaluation of DNA damage, toxicity and functional impairment in human mesenchymal stem cells. Toxicol. Lett..

[26] Gojova A., Guo B., Kota R.S., Rutledge J.C., Kennedy I.M., Barakat A.I. (2007). Induction of inflammation in vascular endothelial cells by metal oxide nanoparticles: Effect of particle composition. Environ. Health Perspect..

[27] Mazzarella G., Ferraraccio F., Prati M.V., Annunziata S., Bianco A., Mezzogiorno A., Liguori G., Angelillo I.F., Cazzola M. (2007). Effects of diesel exhaust particles on human lung epithelial cells: An in vitro study. Respir. Med..

[28] Danielsen P.H., Cao Y., Roursgaard M., Møller P., Loft S. (2015). Endothelial cell activation, oxidative stress and inflammation induced by a panel of metal-based nanomaterials. Nanotoxicology.

[29] Hackenberg S., Scherzed A., Technau A., Kessler M., Froelich K., Ginzkey C., Koehler C., Burghartz M., Hagen R., Kleinsasser N. (2011). Cytotoxic, genotoxic and pro-inflammatory effects of zinc oxide nanoparticles in human nasal mucosa cells in vitro. Toxicol. In Vitro.

[30] Ma H., Williams P.L., Diamond S.A. (2013). Ecotoxicity of manufactured ZnO nanoparticles—A review. Environ. Pollut..

[31] Shalini D., Senthilkumar S., Rajaguru P. (2018). Effect of size and shape on toxicity of zinc oxide (ZnO) nanomaterials in human peripheral blood lymphocytes. Toxicol. Mech. Methods.

[32] Yin H., Casey P.S., McCall M.J., Fenech M. (2015). Size-dependent cytotoxicity and genotoxicity of ZnO particles to human lymphoblastoid (WIL2-NS) cells. Environ. Mol. Mutagen..

[33] Yang H., Liu C., Yang D., Zhang H., Xi Z. (2009). Comparative study of cytotoxicity, oxidative stress and genotoxicity induced by four typical nanomaterials: The role of particle size, shape and composition. J. Appl. Toxicol..

[34] Bihari P., Vippola M., Schultes S., Praetner M., Khandoga A.G., Reichel C.A., Coester C., Tuomi T., Rehberg M., Krombach F. (2008). Optimized Dispersion of Nanoparticles for Biological In Vitro and In Vivo Studies. Part. Fibre Toxicol..

[35] Koch S., Kessler M., Mandel K., Dembski S., Heuzé K., Hackenberg S. (2016). Polycarboxylate ethers: The key towards non-toxic TiO_2_ nanoparticle stabilisation in physiological solutions. Colloids Surf. B Biointerfaces.

[36] Mosmann T. (1983). Rapid colorimetric assay for cellular growth and survival: Application to proliferation and cytotoxicity assays. J. Immunol. Methods.

[37] Froelich K., Steussloff G., Schmidt K., Ramos Tirado M., Technau A., Scherzed A., Hackenberg S., Radeloff A., Hagen R., Kleinsasser N. (2013). DiI labeling of human adipose-derived stem cells: Evaluation of DNA damage, toxicity and functional impairment. Cells Tissues Organs.

[38] Pittenger M.F., Mackay A.M., Beck S.C., Jaiswal R.K., Douglas R., Mosca D.J., Moorman M.A., Simonetti D.W., Craig S.M., Marshak D.R. (1999). Multilineage potential of adult human mesenchymal stem cells. Science.

[39] Nöth U., Osyczka A.M., Tuli R., Hickok N.J., Danielson K.G., Tuan R.S. (2002). Multilineage mesenchymal differentiation potential of human trabecular bone-derived cells. J. Orthop. Res..

[40] Jaiswal N., Haynesworth S.E., Caplan A.I., Bruder S.P. (1997). Osteogenic differentiation of purified, culture-expanded human mesenchymal stem cells in vitro. J. Cell. Biochem..

